# RA signaling pathway combined with Wnt signaling pathway regulates human-induced pluripotent stem cells (hiPSCs) differentiation to sinus node-like cells

**DOI:** 10.1186/s13287-022-03006-8

**Published:** 2022-07-18

**Authors:** Lin Yin, Feng-yuan Wang, Wei Zhang, Xi Wang, Yan-hong Tang, Teng Wang, Yu-ting Chen, Cong-xin Huang

**Affiliations:** 1 Department of Cardiology, Renmin Hospital of Wuhan University, 238 Jiefang Road, Wuchang, Wuhan, 430060 Hubei People’s Republic of China; 2 Cardiovascular Research Institute, Wuhan University, Wuhan, 430060 People’s Republic of China; 3 Hubei Key Laboratory of Cardiology, Wuhan, 430060 People’s Republic of China

**Keywords:** Biological pacemaker, Retinoic acid, Human-induced pluripotent stem cells

## Abstract

**Background:**

The source of SAN is debated among researchers. Many studies have shown that RA and Wnt signaling are involved in heart development. In this study, we investigated the role of retinoic acid (RA) and Wnt signaling in the induction of sinus node-like cells.

**Methods:**

The experimental samples were divided into four groups: control group (CHIR = 0), CHIR = 3, RA + CHIR = 0 andRA + CHIR = 3. After 20 days of differentiation, Western blot, RT-qPCR, immunofluorescence and flow cytometry were performed to identify sinus node-like cells. Finally, whole-cell patch clamp technique was used to record pacing funny current and action potential (AP) in four groups.

**Results:**

The best intervention method used in our experiment was RA = 0.25 µmol/L D5-D9 + CHIR = 3 µmol/L D5-D7. Results showed that CHIR can increase the expression of ISL-1 and TBX3, while RA mainly elevated Shox2. Immunofluorescence assay and flow cytometry further illustrated that combining RA with CHIR can induce sinus node-like cells (CTNT^+^Shox2^+^Nkx2.5^−^). Moreover, CHIR might reduce the frequency of cell beats, but in conjunction with RA could partly compensate for this side effect. Whole cell patch clamps were able to record funny current and the typical sinus node AP in the experimental group, which did not appear in the control group.

**Conclusions:**

Combining RA with Wnt signaling within a specific period can induce sinus node-like cells.

**Supplementary Information:**

The online version contains supplementary material available at 10.1186/s13287-022-03006-8.

## Background

Many signaling pathways (including bone morphogenetic protein (BMP) signaling pathway, Wnt signaling pathway and Retinoic acid (RA) signaling) regulate embryonic sinoatrial node (SAN) development, which is instructive for generating biological pacemakers [1–4]. With the establishment of human-induced pluripotent stem cell (hiPSC) lines and the tracer technology, many scientists have imitated SAN development to induce sinus node-like cells in vitro by adding several small molecular substances [5–8].

Selecting RA and Wnt signaling pathways as experimental subjects is theory-based. Firstly, RA is expressed in the venous pole of the posterior second heart field and regulates the left–right asymmetry of the stages of cardiac development [9–11]. Secondly, a study by Protze SI showed that RA can enhance the phenotype of biological pacemakers. However, due to the complexity of the protocol of that study, it is necessary to find a simpler method of pacemaker generation [8]. Thirdly, Ren' study illustrated that canonical Wnt5b signaling directed outlying Nkx2.5^+^ mesoderm into pacemaker cardiomyocytes [7]. Finally, CHIR synergizes with RA during cardiac development, while no study has elaborated their effects on the formation of a biological pacemaker.

The main aim of our experiment was to determine whether combining RA and Wnt signaling pathways can help to induce sinus node-like cells and the biological mechanism underlying the induction.

## Materials and methods

### Cell source

Human-urothelium-derived-induced pluripotent stem cells (U1) and human embryonic pluripotent stem cells (H9) were purchased from CELLAPY Corporation.

### Cell culture and identification

#### Preparation before cell culture

In every six-well culture plate, 1 ml of coated medium (Aliquot Matrigel (Cat. no. 354277; Corning) + Knockout DMEM + 1% penicillin/streptomycin) was added. The liquid was quickly released at the center of the bottom of the culture plate, which was shaken immediately after adding the medium to each well, allowing the liquid to evenly spread throughout the culture plate. The whole process was performed quickly to avoid rewarming of the Matrix gel. A plate coated with Matrigel can be left in a refrigerator at 4℃ for no more than one week.

#### Cell passage and culture

When the cells reached 80–90% confluence, they were passaged. Versene (Cat. no. 15040066; Gibco) digestion solution was added to digest at 37℃ for 6–8 min. When the cells turned spherical and the intercellular gaps became larger, the digestion solution was aspirated, and 1 ml of subculture medium was added to each well to stop digestion. The passage ratio was generally 1:5 to 1:10. The cells in the culture plates were shaken before and after for uniform distribution. The medium was removed and replaced daily by a fresh mTeSR1 medium (Cat. no. 85850; STEMCELL Technologies). On the first day, ROCK inhibitor Y27632 (Cat. no. 72302; STEMCELL Technologies) was added to the culture medium to facilitate cell survival.

#### Cell differentiation

Based on previous protocols and former experience in our laboratory, hiPSCs or hESCs were differentiated into cardiomyocytes by manipulating Wnt/β‐catenin signaling [12, 13]. Briefly, at the beginning of cardiac differentiation (Day 0), hiPSCs or hESCs were cultured in the basic differentiation media (containing Roswell Park Memorial Institute [RPMI]-1640 medium, B27 minus insulin (Cat. no. A189560; Gibco), L-ascorbic acid 2‐phosphate sesqui-magnesium salt hydrate (Cat. no. A8960; Sigma-Aldrich)) combined with Wnt/β-catenin signaling activator CHIR99021 (Cat.no.2052; STEMCELL Technologies) for 48 h. After two days, the medium was replaced by a basic differentiation medium for 24 h. Then, the medium was replaced by a cardiac mesoderm induction medium that consisted of RPMI-1640/B27 minus insulin with Wnt pathway inhibitor IWR-1 (Cat. no. 72562; STEMCELL Technologies) for another 48 h. On day 5, the medium was removed and the cells were maintained in RPMI-1640/B27 minus insulin without any additional factors for two days. After that, the cell cultures were changed to RPMI-1640/B27 supplement with insulin (Cat. no. 17504044; Gibco) for the next few days and refreshed every other day. The cell morphology and cell pulsation in each stage of differentiation were observed and photographed by a microscope, and the markers of cells at each stage of differentiation were identified. D20 was the end observation time.

### Reverse transcription-quantitative polymerase chain reaction (RT-qPCR)

Total RNA was extracted from the differentiated hiPSCs or hESCs on D20 using TRIzol® reagent (Invitrogen). Quantitative PCR was performed to evaluate the mRNA expression of SRY-box transcription factor 2(SOX2), octamer‐binding protein 4 (OCT-4), mesoderm specific T-box transcription factor Brachyury(BryT), mesoderm posterior BHLH transcription factor 1(Mesp1), cardiac troponin T(cTNT), gene encoding atrial natriuretic peptide(NPPA), sodium voltage-gated channel alpha subunit 5(SCN5A), myosin, light polypeptide 2(MYL-2), human short stature homeobox 2(Shox2), insulin gene enhancer binding protein 1(ISL-1), T-box 18(TBX18), TBX3, TBX5, hyper-polarization activated cyclic nucleotide-gated potassium channel 4(HCN4), paired-like homeodomain transcription factor 2(Pitx2) and related iron channels as well as connexion. Isolated RNA (2 µg) was converted into cDNA using a First Strand cDNA Synthesis Kit (Toyobo Life Science) as follows: 42 °C for 2 min, 37 °C for 15 min, 85 °C for 5 min, and 4 °C for 10 min. The primers used for PCR amplification were synthesized by Invitrogen Biotechnology (Thermo Fisher Scientific) and are presented in Table 1. RT-qPCR was performed using the Step-One™ Real-Time PCR system (Life Technologies, Carlsbad, CA, USA). The reactions were then conducted using the SYBR® Premix Ex Taq TM II (Takara Bio, Japan). Protocol was as follows: pre-denaturation at 95 °C for 3 min, denaturation at 95 °C for 30 s, annealing at 58 °C for 10 s, and a final extension at 72 °C for 30 s, a total of 40 cycles. Semi-log amplification curves were analyzed using the 2^−ΔΔCt^ comparative quantification method and the expression of each gene was normalized to β-actin. PCR analyses were repeated at least three times to verify results.

**Table 1:** Polymerase chain reaction primers used in this study

Gene		Primer(5’—3’)
TBP	Forward	TGAGTTGCTCATACCGTGCTGCTA
	Reverse	CCCTCAAACCAACTTGTCAACAGC
SOX2	Forward	AGCTACAGCATGATGCAGGA
	Reverse	GGTCATGGAGTTGTACTGCA
OCT-4	Forward	AGCGAACCAGTATCGAGAAC
	Reverse	TTACAGAACCACACTCGGAC
BryT	Forward	TGTCCCAGGTGGCTTACAGATGA
	Reverse	GGTGTGCCAAAGTTGCCAATACA
Mesp1	Forward	AGCCCAAGTGACAAGGGACA
	Reverse	AAGGAACCACTTCGAAGGTGC
cTNT	Forward	TTCACCAAAGATCTGCTCCTCGCT
	Reverse	TTATTACTGGTGTGGAGTGGGTGTGG
NPPA	Forward	GGGTCTCTGCTGCATTTGTGTCAT
	Reverse	AGAGGCGAGGAAGTCACCATCAAA
SCN5A	Forward	TGCTGCTCTTCCTCGTCATGTTCA
	Reverse	TGTTGGCGAAGGT CTGGAAGTTGA
MYL-2	Forward	TGTCCCTACCTTGTCGTTAG
	Reverse	ATTGGAACATGGCCTCTGGAT
TBX5	Forward	ACAAAGTGAAGGTGACGGGCCTTA
	Reverse	ATCTGTGATCGTCGGCAGGTACAA
TBX3	Forward	TTGAAGACCATGGAGCCCGAAGAA
	Reverse	CCCGCTTGTGAAACTGATCCCAAA
Nkx2.5	Forward	CCACGCCCTTCTCAGTCAAA
	Reverse	TCAGGCTTTCTTTTCGGCTCTA
Shox2	Forward	ATCGCAAAGAGGATGCGAAAGGGA
	Reverse	TTCCAGGGTGAAATTGGTCCGACT
TBX18	Forward	TTAACCTTGTCCGTCTGCCTGAGT
	Reverse	GTAATGGGCTTTGGCCTTTGCACT
ISL-1	Forward	GAAGGTGGAGCTGCATTGGTTTGA
	Reverse	TAAACCAGCTACAGGACAGGCCAA
HCN4	Forward	TCTTCCTCATTGTGGAGACACGCA
	Reverse	TGAGGATCTTCGTGAAGCGGACAA
Cx45	Forward	AGAGCAGAGCCAACCCAAACCTAA
	Reverse	GCCAGCAACTGCAGCACATAGATT
Pitx2	Forward	GCAGCGGACTCACTTTACCA
	Reverse	TAAGGTTGGTCCACACAGCG
KCNJ2	Forward	TGGTGGTGTTCCAGTCAATC
	Reverse	CTCGTTTCTCTTCTTTGGCTTTG
Cacna1c	Forward	CCAACCTGGAACGAGTGGAATA
	Reverse	CACTAAAAAGCCCCACAACCAC
Cacna1g	Forward	CGACCGTGAAATGCCTGACT
	Reverse	CTCGGGCTGCTCGTGGTATT
NCX	Forward	CCTCCACTGCCACTGTAACTATTT
	Reverse	TCTTCTCACTCATCTCCACCAGG
Cx43	Forward	GATGAGGAGTCTGCCTTTCGTTGT
	Reverse	AGAAGCGCACATGAGAGATTGGGA
miR-106b	Forward	CGGGGCTAAAGTGCTGACA
	Reverse	GAGCAGCAAGTACCCACAGT
miR-17–92	Forward	CCTGTTGAGTTTGGTGGGGA
	Reverse	CAAATCTGACACGCAACCCC
miR-1	Forward	TTCGAGCAACTGGGCAAGAC
	Reverse	GACCTTGTTGGCGTGCTT
BMP4	Forward	TTCTCGACTCCGGGGAACAT
	Reverse	AACGACCATCAGCATTCGGT

### Western blot analysis

The differentiated hiPSCs on D20 were plated on 12-well culture dishes. The cells were harvested using RIPA lysis buffer (Beyotime Institute of Biotechnology, Haimen, China). Equal amounts of proteins were loaded onto a gel for sodium dodecyl sulfate–polyacrylamide gel electrophoresis (SDS-PAGE), and the separated proteins were transferred onto a nitrocellulose membrane, and incubated with primary antibodies against HCN4 (ab32675; Abcam, Cambridge, MA, USA), TBX18(ab115262; Abcam, Cambridge, MA, USA), ISL-1(abs132916; Abcam, Cambridge, MA, USA) TBX3(ab154828; Abcam, Cambridge, MA, USA), TBX5(Gene-Tex; Cat No. GTX113849), α-tubulin (GB11200; Service-bio, Wuhan, China), GAPDH antibody (Abcam; Cat. no. ab37168) and Shox2(ab55740; Abcam, Cambridge, MA, USA) overnight at 4 °C. The primary antibodies were detected by incubating the membrane with secondary antibodies for 1 h, and then enhanced chemiluminescence detection (ECL; Beyotime Institute of Biotechnology) was performed. The level of α-tubulin or GAPDH was considered to normalize the signal intensities. The Western blot assay was repeated at least three times to verify the results.

### Immunostaining studies

The differentiated hiPSCs on D20 were placed on gelatin-coated coverslips in 12-well culture dishes. The cell cultures were washed with PBS and fixed with 4% paraformaldehyde, followed by permeabilization with 0.1% Triton X-100 for 15 min and blocked using bovine serum albumin solution for 30 min at room temperature. Then the cells were incubated with the primary antibodies anti- cTNT (Abcam, cat. no. ab8295, 1:400) and anti-Shox2 (1:800) overnight at 4 °C. The secondary antibodies Cy3-conjugated Affinipure goat anti-rabbit IgG (Cat. no.111-165-003, 1:300) and FITC-AffiniPure F(ab’)2 Fragment goat anti-mouse IgG (Cat. no.115–096-006,1:400) (both from Jackson Immuno-Research Laboratories, Inc., West Grove, PA, USA) were incubated for another 1 h. 4’,6-Diamidino-2-phenylindole (DAPI) was used to visualize the nuclei. The cells were observed under a fluorescent microscope (Leica, Wetzlar, Germany). We randomly selected three visual fields from three different cell isolates to observe the positive rates.

### Patch clamp

Whole-cell electrophysiology was performed as described. The cells were plated on gelatin-coated coverslips in 24-well culture dishes. The whole cell patch-clamp technique was used to record the funny current (I_f_ current) after 2–4 days of re-plating. We selected smooth and plump cells to record the current. The impedance of the fluid-filled electrode was 5 to 8MΩ. The experiments were performed using an Axon patch-clamp amplifier 700B (Molecular Devices, Sunnyvale, CA, USA). A digital 700AD/DA converter and 6.0.4 p-Clamp (both from Axon Instruments, Union City, CA, USA) were used for recording and analyzing the data.

I_f_ current and AP bath solution has the following components (in mmol/l): 140 NaCl, 5.4 KCl, 1.0 MgCl2, 1.8 CaCl2, 1.0 BaCl2, 5.5 HEPES, 5.0 glucose (pH 7.4); the pipette solution contained the following (in mmol/l): 20 KCl, 125 K-gluconate, 1.0 MgCl2, 5.0 NaCl, 10 HEPES, 5 K2ATP (pH 7.2). The whole cell recording mode was used to record I_f_ current. The Clampex program was used to analyze the sample. The sampling frequency was 10 kHz, and the filtering rate was 5 kHz. I_f_ currents were recorded by holding the resting membrane potential at − 40 mV, then stepping to a test voltage of − 140 mV for 2 s with a step of 10 mV in each sweep.

### Flow cytometry analysis

The differentiated hiPSCs or hESCs on D20 were digested with 0.25% trypsin and 0.5 mM ethylenediaminetetraacetic acid to form a single-cell suspension, washed with PBS, and centrifuged at 300 g for 5 min. After centrifugation, the cells were fixed with 4% paraformaldehyde for 30 min at room temperature, then permeabilized and blocked by 100ul blocking solution (PBS + 5% FBS + 0.2% trelaton + 0.1% Triton X-100) overnight at 4 °C. The cells were incubated with mouse anti-cTNT or mouse anti-Shox2 (Immunoway Cat no.YT6364) and rabbit anti-Nkx2.5(E1Y8H#8792; Cell Signaling Technology, Massachusetts, USA) in the dark for 1 h at room temperature, washed with PBS twice and followed by incubation with the corresponding secondary antibodies for another 30 min. Finally, the cells were resuspended in PBS before performing a flow cytometer assay (Beckman CytoFLEX).

### Statistical data analysis

GraphPad Prism7 was used for data analysis. The reported data were expressed as the means ± SEM. The statistical significance of the differences between groups was determined using the one-way ANOVA. All data were subjected to formal tests for normality. Data not exhibiting a normal distribution were evaluated by non-parametric tests. P < 0.05 was considered to indicate a statistically significant difference.

## Results

### Differentiation of hiPSCs into CMs

**Fig. 1:**
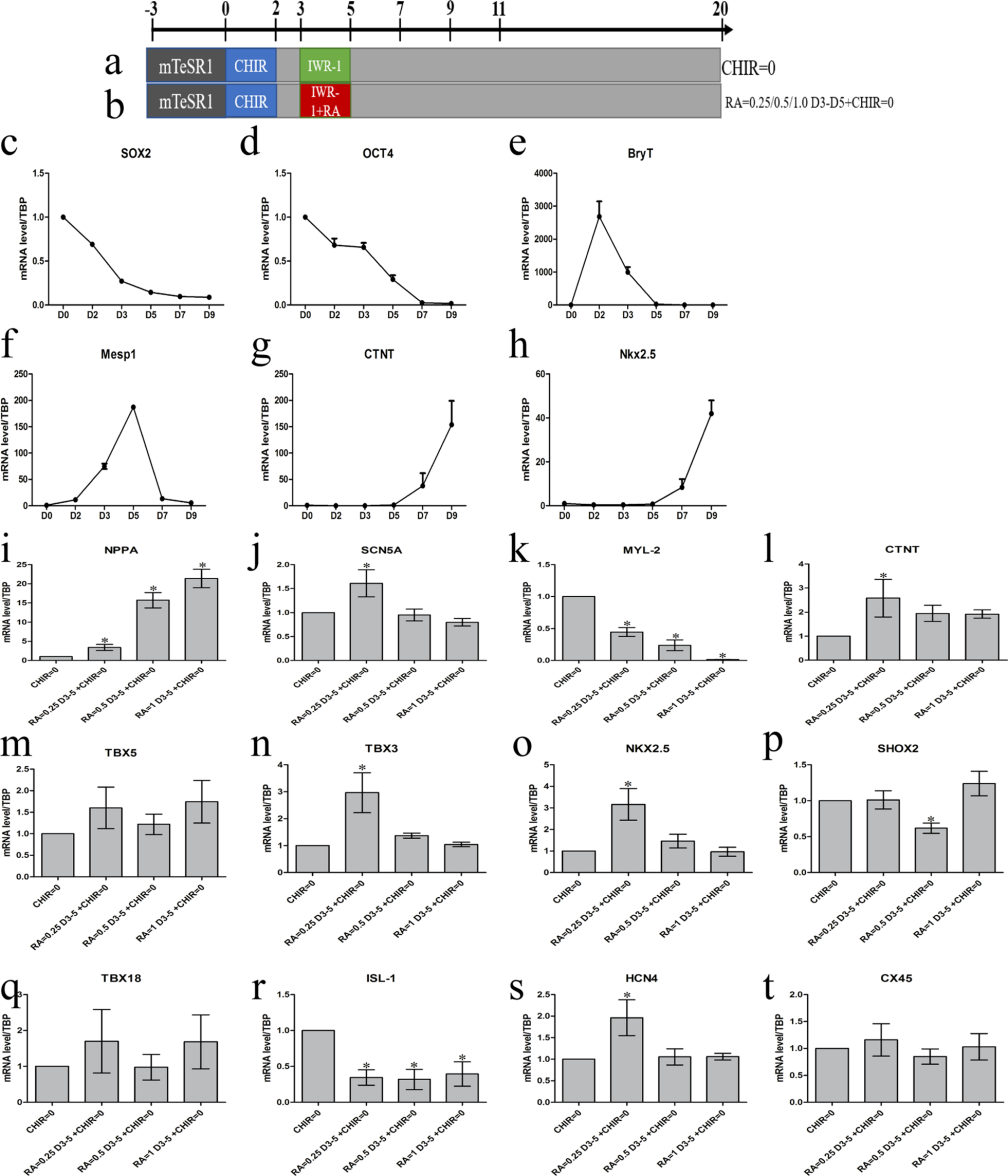
Differentiation of hiPSCS into CMs and exploration of the optimal RA intervention time. Data points present as means ± SEM (*n* = 1). **a** Schematic of the experimental protocol for the differentiations of cardiomyocybytes from hiPCs with small molecule modulators of Wnt signaling. CHIR (CHIR99021) and IWRI, agonists, and inhibitors of the Wnt pathway, respectively. **b** Schematic of the experimental protocol for RA = 02.4/0.5/1.0umol at days D3-D5 to include hiPSCs differentiation, **c**–**h** Samples of mRNA were collected and measured from differentiating cells between day0–9 using qRT-PCR and normalized to TBP expression. **i**–**t** The4 transcriptional level of the central atrial muscle cell marker and pacemaker markers. Data points present as means ± SEM (n = 1). *p < 0.05 compared to control group (CHIR = 0 group). RT-qPCR, quantitative reverse transcriptase-polymerase chain reaction

In this study, following a previously designed protocol in our laboratory, we used Wnt signaling activator CHIR and Wnt signaling inhibitor IWR-1 to induce cardiac differentiation (Fig. 1a). The pluripotency marks (SOX2 and OCT4) showed the highest expression on D0 (Figs. 1c, d). Expression of the mesoderm-specific T-box transcription factor Brachyury (BryT) was observed on from D1 to D3 of differentiation, which indicated that the hiPSCs had undergone mesodermal induction (Fig. 1e). Mesp1, the characteristic marker of the cardiac mesoderm stage increased from D3 to D5 (Fig. 1f). Early cardiomyocyte makers (Nkx2.5 and CTNT) were detected as early as on D7 of differentiation (Fig. 1g, h).

### The optimal RA intervention time

Previous studies have found that from D3to D5, RA (HY-14649; MedChemExpress) intervention can enhance the pacemaker phenotype [8]. Therefore, our experiment initially followed this protocol to add RA = 0.25/0.5/1.0 µmol/L at days D3-D5 to induce the differentiation of hiPSCs (Fig. 1b). We found that the atrial muscle cell marker differentiated with RA at D3-D5 elevated relative to its level in the control group (CHIR = 0 group), but the levels of the pacing markers, such as Shox2 and TBX18, almost had no change (Fig. 1i–t). Several related articles about RA and cardiac embryonic development and showed that simple supplementation with RA at the embryonic stage E8.5-E14.5 could remedy the development of the SAN in RALDH2 knockout mice [14]. Moreover, a study by Ren showed that CHIR = 3 µmol/L D5-D7 can increase the generation of hiPSC-pacemaker cardiomyocytes [7]. Based on the results of those studies, we adjusted the protocol. The RA intervention period changed to RA = 0.25 µmol/L D5-D7, D7-D9, D9-D11, D5-D11, respectively, combined with CHIR = 3 µmol/L D5-D7 or not (Additional file 1: Figure S1a). RNA results showed that both D7-D9 and D9-D11 groups could improve the expressions of pacing-related transcription factors and the pacing proteins HCN4 (Additional file 1: Figure S1b). To further determine the optimal experimental protocol of RA intervention, we expanded the RA intervention time to D5-D9, D7-D9 and D7-D11 (Additional file 1: Figure S1c). As the results showed that the mRNA levels of Shox2 and HCN4 in D5-D9 increased significantly compared to their levels in the other groups (Additional file 1: Figure S1d). Then we used RA = 0.25 µmol/L D5-D9 + CHIR = 3 µmol/L D5-D7 as the optimal RA intervention time.

### The optimal RA intervention concentration

Based on the optimal intervention times described above, we further investigated the optimal intervention concentrations of RA. We used three concentration gradients of RA = 0.25, 0.5, and 1.0 µmol/L for observation. The results showed that pacing-related transcription factors and proteins were the most obvious at RA = 0.25 µmol/L group, and there was no significant effect on the expression of CTNT (Additional file 2: Figure S2a, S2b).

### RA promoted the differentiation of hiPSCs into sinus node-like cells

**Fig. 2:**
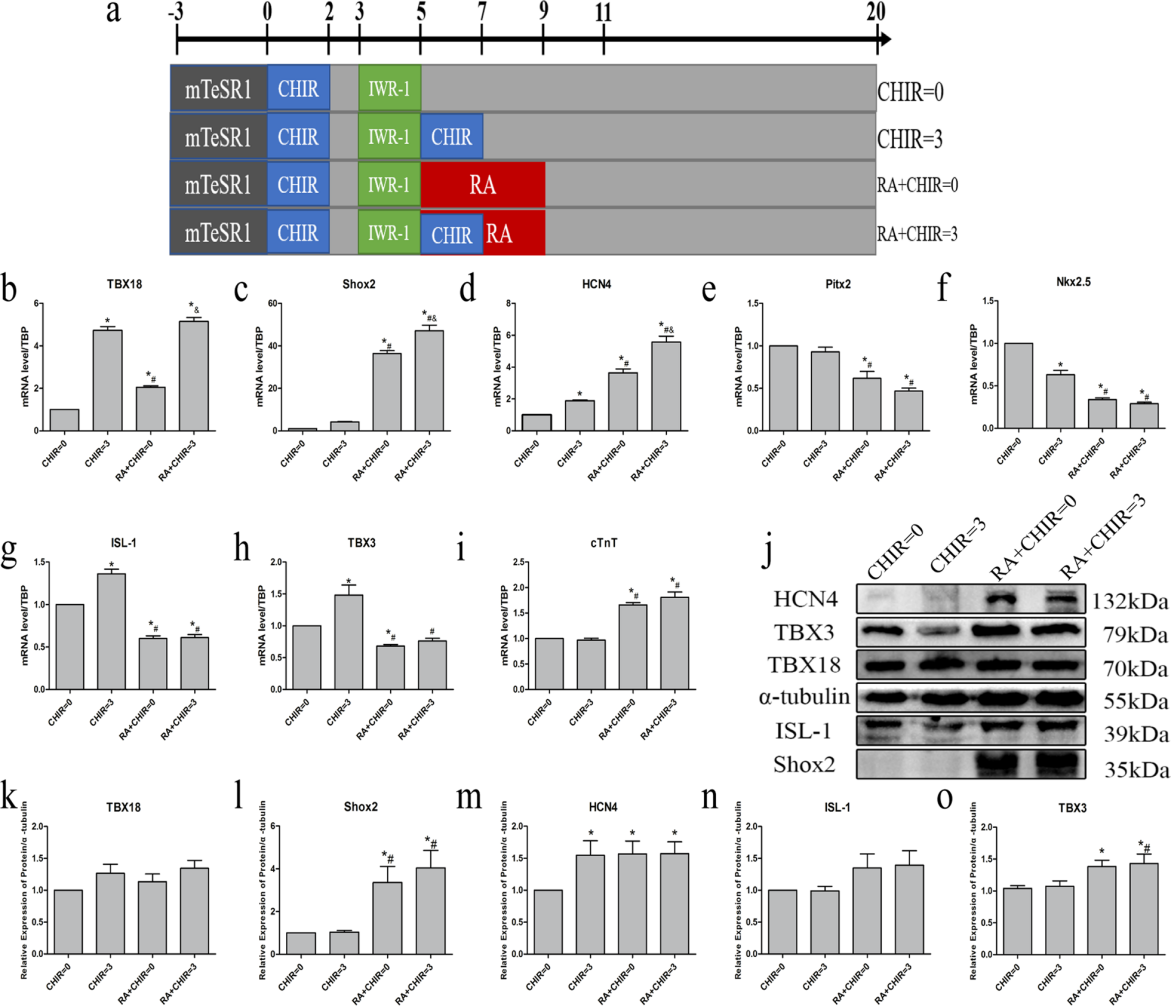
The expression of genes and proteins related to cardiac pacemaker cells in four groups. **a** Schematic of the experimental protocol for RA in four groups. **b**–**i** The mRNA level of the pacemaker markers. **j**–**o** The protein expressions of the pacemaker markers. Data points present as means ± SEM (*n* = 1). *p < 0.05 compared to CHIR = 0 group. ^#^*p* < 0.05 compared to CHIR = 3 group. ^&^*p* < 0.05 compared to RA + CHIR = 0 group

Combined with the above experiments, we finally determined that the best intervention was RA = 0.25 µmol/L D5-D9 + CHIR = 3 µmol/L D5-D7. Then we divided the samples into four groups: CHIR = 0, CHIR = 3, RA + CHIR = 0, and RA + CHIR = 3(Fig. 2a). The results of Fig. 2b showed that both CHIR and RA increased the mRNA level of TBX18, while CHIR had stronger influence than RA. Experimental studies illustrated that administering RA alone can increase the mRNA level of Shox2 and the pacing protein HCN4, and reduce the level of Nkx2.5 and Pitx2. The effects of the combined treatment of RA and CHIR were more prominent, and the results were statistically significant (Fig. 2c–f). In addition, the results of the RT-qPCR analysis demonstrated that CHIR can increase the mRNA level of Tbx3 and ISL-1, but in RA + CHIR = 0 and RA + CHIR = 3 group, the levels of both TBX3 and ISL-1 decreased (Fig. 2g, h). Surprisingly, RA could dramatically enhance the level of CTNT (Fig. 2i). The expressions of the TBX18 and ISL-1 proteins in four groups were similar (Fig. 2j, k, l). RA substantially enhanced the expression of Shox2 and slightly increased TBX3 (Fig. 2j, m, n). The HCN4 level increased in the other three groups compared to that in the group with CHIR = 0 (Fig. 2j, o).

### RA rose the frequency of beats within the cardiac cell mass

**Fig. 3:**
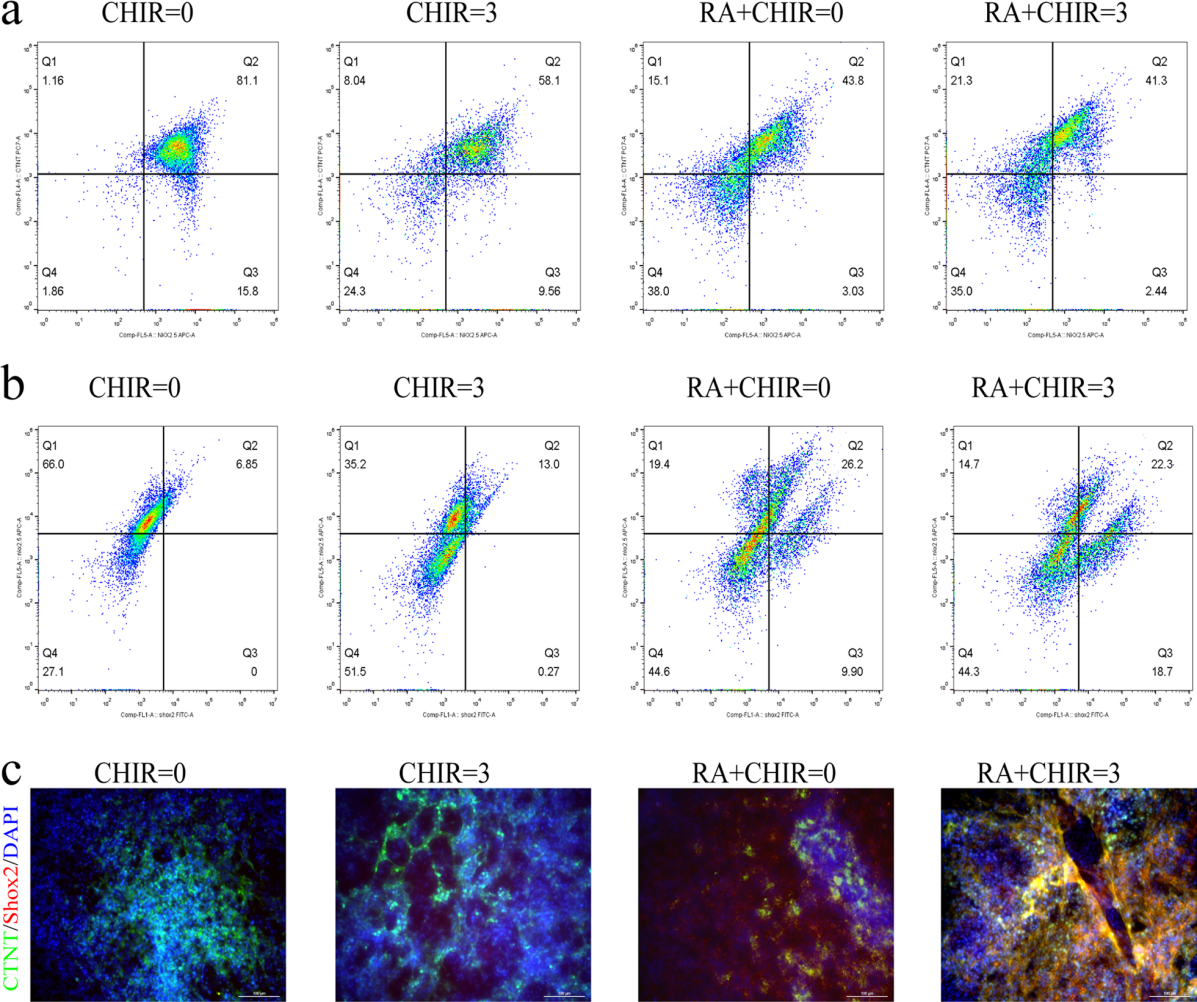
Ra promoted the differentiation of hiPSCs inti pacemaker cells. **a** The pacemaker cells rate marked by both CTNT positive and Nkx2.5 negative (cTNT^+^Nkx2.5^−^) in four groups detected by flow cytometry (*n* = 4). **b** The pacemaker cells rate marked by both Shox2 positive and Nkx2.5 negative (Shox2^+^Nkx2.5.^−^) in four groups detected by flow cytometry (*n* = 4). **c** Cells differentiated from four groups were stained for cTNT (green), Shox2 (red) and DAPI (blue). Magnification, × 200, *n* = 4

**Fig. 4:**
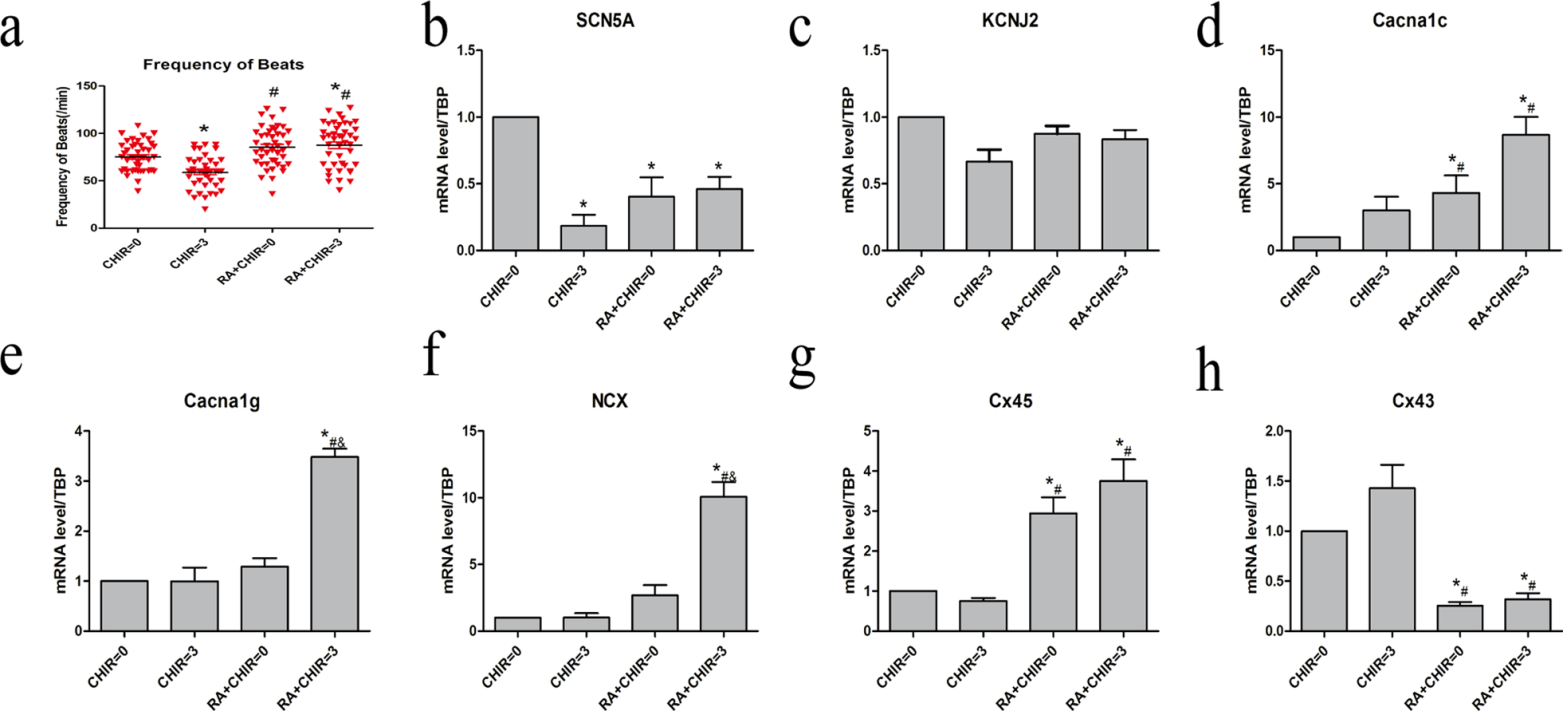
The frequency of beats and the expression of ionic channels in four groups. **a** The frequency of beats in four groups. **b**–**h** The mRNA level of ionic channels in four groups. Data points present as means ± SEM (*n* = 5). *p < 0.05 compared to CHIR = 0 group. ^#^*p* < 0.05 compared to CHIR = 3 groups. ^&^*p* < 0.05 compared to RA + CHIR = 0 group

The results of the flow cytometry and immunofluorescence assays further illustrated that RA combined with CHIR significantly increased the proportion of hiPSC-pacemaker cardiomyocytes (CTNT^+^Shox2^+^Nkx2.5^−^), where CHIR reduced the expression of NKx2.5, while RA mainly increased Shox2 and slightly reduced Nkx2.5 (Fig. 3). Although CHIR can increase the proportion of pacing cells, it partially decreased the frequency of cell beats, and combined with RA can compensate for this side effect (Fig. 4a, Additional file 3 : Video S1, Additional file 4: Video S2, Additional file 5: Video S3, Additional file 6: Video S4).

### Effect on the expression of ionic channels

We further examined the effects of RA on the expressions of the relevant ionic channels. RA and CHIR interventions reduced the expression of sodium channels without significantly affecting potassium channels (Fig. 4b, c). Additionally, RA, along with CHIR, significantly amplified the transcription level of the calcium ion channels, cardiac sodium-calcium exchanger (NCX) and connexin 45 (Cx45), and reduced the expression of Cx43 (Fig. 4d–h), which might be the ion mechanism by which RA increases the frequency of beating.

### Recorded funny current and action potential in four groups

**Fig. 5:**
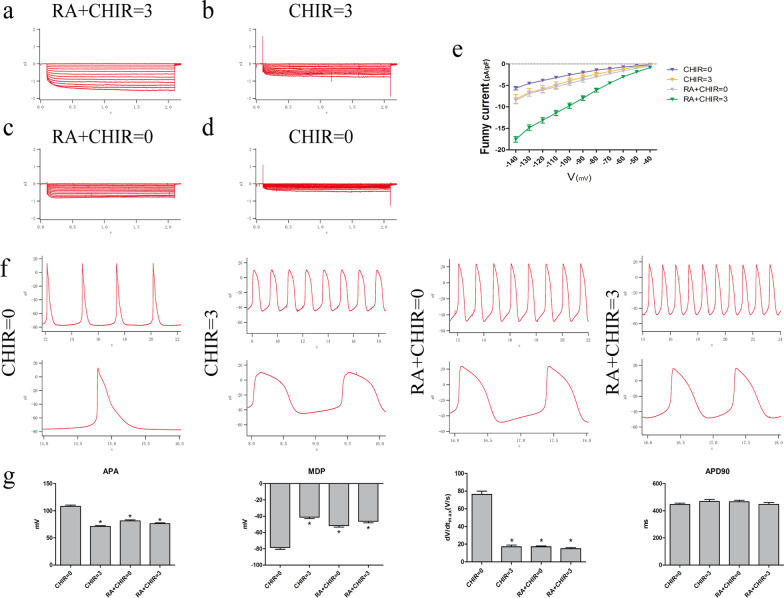
I_f_ current and typical action potential (AP) of four groups. **a**–**d** I_f_ Current was detected in four groups using the patch clamp technique (*n* = 28). **e** Current density–voltage relationships in four groups (*n* = 28). **f** AP was detected in four groups. **g** APA, MDP level, 0-phase depolarization rate (dV/dt) and APD of four groups.*p < 0.05 compared to CHIR = 0 groups. APA, action potential amplitude; action potential duration; MDP, maximum diastolic potential

We investigated the electrophysiological properties of the four groups of cells using the patch-clamp technique. The results showed that the funny current could be recorded in the later three groups (CHIR = 3, RA + CHIR = 0, and RA + CHIR = 3). The highest current was recorded in the RA + CHIR = 3 group, and the current decreased with the reduction of the given voltage stimulation (Fig. 5a–e). The morphology AP of the control group was similar to that of working cardiomyocytes, while the typical sinus node AP was recorded in the other three groups (Fig. 5f). Furthermore, the parameters of AP showed that AP amplitude (APA), the maximum diastolic membrane potential (MDP) level and 0-phase depolarization rate (dV/dt) in the later three groups were smaller than that in the CHIR = 0 group, while AP duration (APD_90_) was not significantly different (Fig. 5g).

### The biological mechanism by which RA promotes pacing cell differentiation

**Fig. 6:**
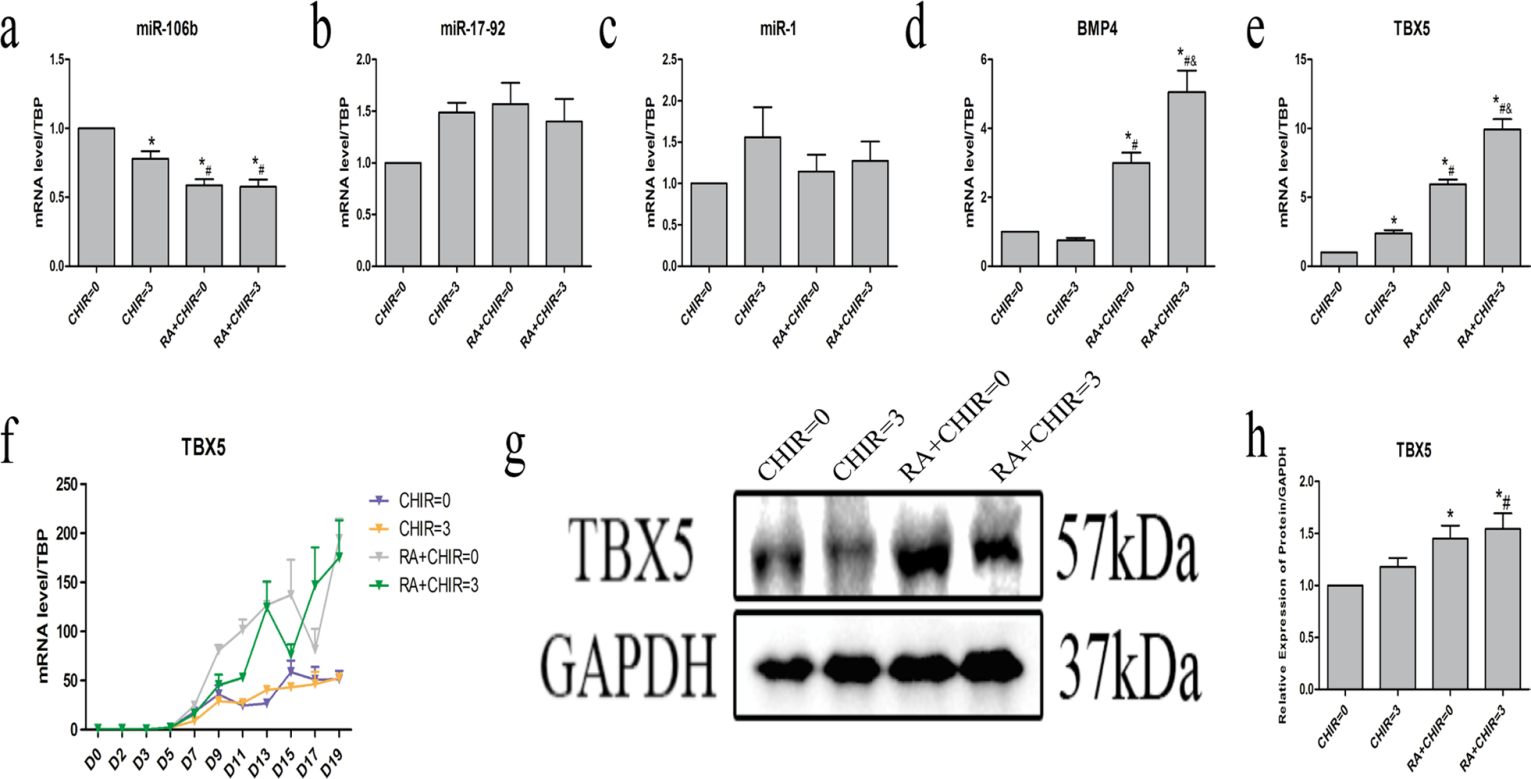
The biological mechanism by which RA promotes pacing cell differentiation. **a**–**e** The mRNA level of the pacemaker markers on D20. **f** The mRNA level of TBX5 on D0–D19. **g**–**h** The protein expression of TBX5. Data points present as means ± SEM (*n* = 5). *p < 0.05 compared to CHIR = 0 group. ^#^*p* < 0.05 compared to CHIR = 3 group. ^&^*p* < 0.005 compared to RA + CHIR = 0 group

**Fig. 7:**
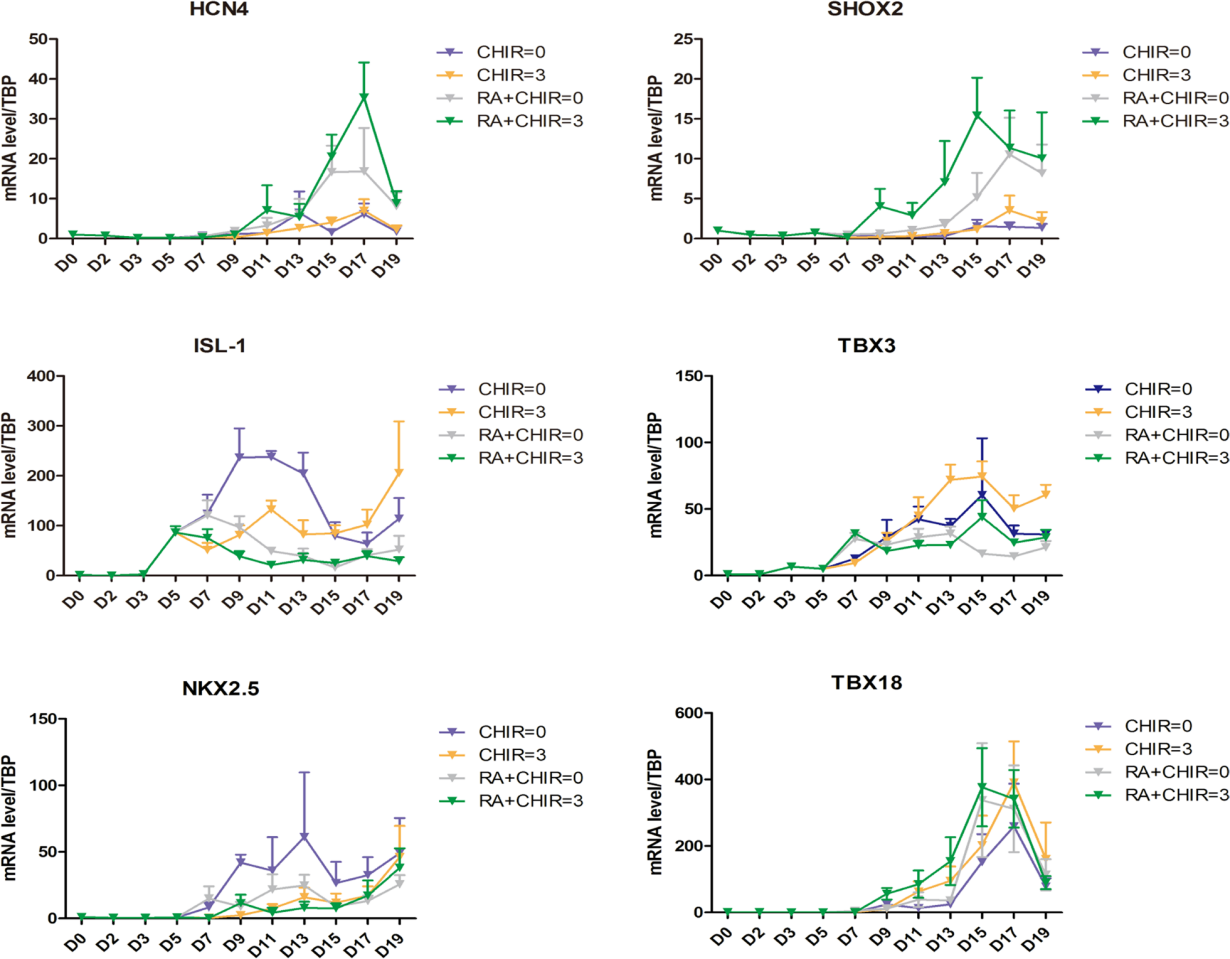
The changes of cardiac pacing-related factors during D0-D19 dynamically

To determine the potential biological mechanism by which RA increased the proportion of pacemaker cells, we analyzed several transcription factors and miRNAs associated with the development of SAN. We found that both RA and CHIR reduced the transcription level of miR-106b, but had no significant effect on the content of miR-17–92 and miR-1 (Fig. 6a–c). Additionally, RA significantly enlarged the levels of TBX5 and BMP (Fig. 6d–e). Dynamic changes of TBX5 from D0 to D19 showed that TBX5 increased continuously from D5 till the final observation (Fig. 6f). The results of Western blot assays demonstrated that RA significantly enhanced TBX5 expression (Fig. 6g, h). We further investigated the changes of cardiac pacing-related factors from D0 to D19 dynamically. We found that RA substantially increased Shox2 from D9 and HCN4 after D13, while CHIR influenced TBX3 after D11 and ISL-1 after D15. Both RA and CHIR reduced Nkx2.5 and slightly increased TBX18 from D9 (Fig. 7).

## Discussion

Shox2 is expressed in the sinus region during mouse embryonic stage E5.5 [15]. Shox2 mutation leads to the death of the embryo at about E11.5, due to a failure of SAN [16]. The phosphorylation of Shox2 can inhibit the expression of Nkx2.5, which prevents the transformation of sinus node cells into working cardiomyocytes [17]. Moreover, cardiac-specific knockout Shox2 gene decreases the heart rate and changes gene expression patterns, such as downregulation of ISL-1and HCN4, and ectopic-expression of Cx40 and Nkx2.5 at the SAN site [18]. Finally, overexpression of Shox2 in embryonic stem cells can induce the formation of pace-like cells and increase the frequency of physiological pacing [19].

The 4q25 site mutations on human chromosome 4 are associated familial atrial fibrillation and are independent risk factors for early-onset atrial fibrillation [20, 21]. Patients carrying this mutation are more likely to have a psychogenic stroke and recurrence after atrial fibrillation ablation [22, 23]. The Pitx2 gene is close to this site and is associated with atrial fibrillation [24, 25]. Pitx2 is widely expressed in the left side of the heart, such as the left atrium, pulmonary vein, and left vena cava, which induces ectopic electrical activity [26]. Wang's study showed that Pitx2 affects two microRNAs (miR-17–92 and miR-106b-25) and indirectly regulates the expression of Shox2 and TBX3, thus causing sinus node dysfunction and increasing the susceptibility to atrial fibrillation [27].

TBX5 is involved in the formation of the entire heart (cardiac conduction system, atrium, ventricles, etc.). Initially, TBX5 is widely expressed in the posterior side of the first and the second heart fields of the heart at the embryonic stage [28]. TBX5 protein can act on the upstream regulatory sites of many transcription factors, such as TBX3, Shox2 and Pitx2 [29]. Moreover, Zhang et al. found that the Hedgehog-TBX5-HCN4 pathway regulates embryonic development of the atrial nodes of the heart, and Raghunathan's study showed that SHT5 (the combined overexpression of three factors, including Shox2, HCN2, and TBX5) can increase the proportion of pacing cells [30]. Additionally, TBX5^+^Nkx2.5^−^ cells can label cardiac sinus node cells, while TBX5^+^Nkx2.5^+^ cells mark the first heart field [31]. Finally, TBX5 is found in the cardiac conduction system in adulthood [32]. TBX3 and TBX5 can jointly mark the cardiac conduction system, and TBX5 knockout mice show abnormal heart rates, such as sinus arrest, AV block and ventricular tachycardia [31, 33].

**Fig. 8:**
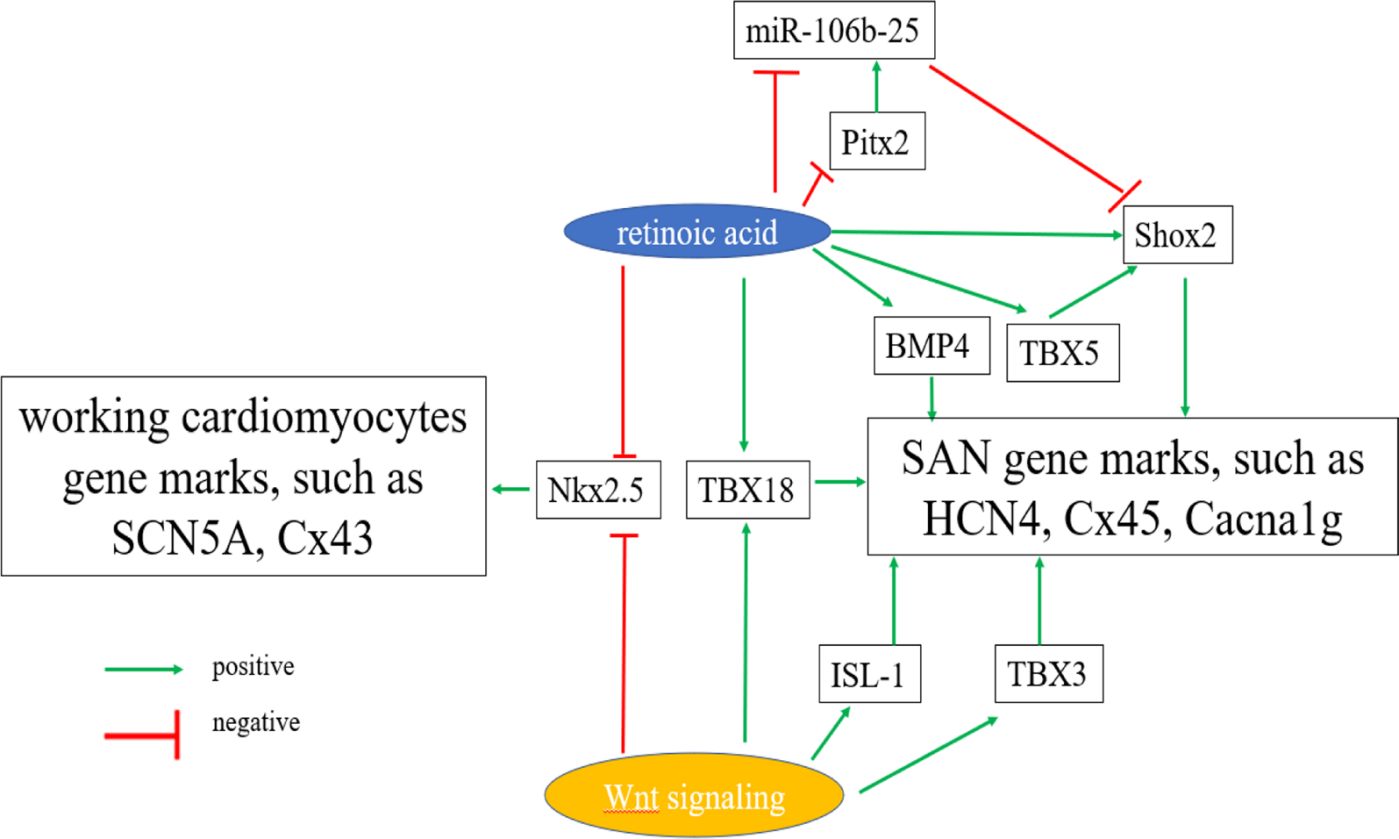
The potential biological of combining Wnt signaling and RA inducing sinus node-like cells

Figure 8 shows possibly potential biological mechanisms of our article: on the one hand, RA can augment the expression of TBX5, Shox2 and HCN4. As mentioned above, TBX5 acts on the upstream regulatory sites of Shox2. On the other hand, RA can also reduce Pitx2 and miR-106b, two upstream inhibitors of Shox2 during the development of SAN. Thus, we inferred that RA-TBX5/Pitx2-Shox2-HCN4 pathway is a possible mechanism by which RA signaling initiates pacemaker differentiation. In addition, we found that RA had a negligible effect on the mRNA level and protein expression of ISL-1 and TBX3, which are mainly enhanced by Wnt signaling. Therefore, we combined both Wnt signaling and RA signaling to generate sinus node-like cells because of their synergistic effects. Moreover, previous studies have shown that BMP signaling specifies cardiac mesoderm toward the pacemaker cells fate, and our study disclosed RA can increase the mRNA level of BMP4 significantly. Finally, RA and CHIR together can slightly regulate TBX18 and Nkx2.5.

The indispensable role of the Wnt signaling pathway in SAN development was shown previously. Using tracer technology, researchers found that the Wnt signaling pathway is continuously expressed in the "third heart field," which is conducive to stage 4 automatic depolarization [34]. Researchers injected Wnt8c into the first and second heart regions and found that the speed of automatic depolarization and pulsating heart rate in stage 4 increased significantly. In addition, the classical wnt5b can directly induce Nkx2.5^+^ cells to sinus node-like cells [7]. Finally, another study suggested that the classical Wnt signaling pathway promotes the differentiation of cardiac precursor cells into sinus node cells, while the non-classical pathway regulates the formation of working cardiomyocytes [35, 36].

Due to the limitations of current technology and the lack of specific markers for SAN, the source of SAN is not clear, although the current view is that the sinus node cells are derived from the right venous sinus [37–39]. Structurally and functionally, SAN is a heterogeneous tissue, i.e., it is a synthesis of many different subpopulations of cells. The sinus node consists of a large "head" within the right jugular vein myocardium and a "tail" distributed along the terminal crest [40]. TBX18 plays a decisive role in the "head" structure, which has a small effect on the electrophysiological function of the sinus node. In contrast, a Tbx3-deficient or Shox2-deficient embryo can form a morphologically normal SAN but a slowed heart rate.

Based on the above results, researchers have used several small molecules to induce sinus node-like cells. Considering that the differentiation process is complex, it is necessary to seek a simpler and more effective differentiation scheme. Additionally, whether sinus node cells induced by those processes belong to the same subtype needs to be elucidated. Ren et al. found that Wnt signaling may initiate pacemaker differentiation by interacting with both ISL-1 and Tbx18 enhancers [7]. Protze et al. showed that BMP can increase the proportion of SANLPCs, while RA enhances the pacemaker phenotype of SANLPCs [8]. Moreover, the "SAN-linked cells" expressing Shox2^+^Nkx2-5^+^ in the transition zone play a crucial role in sinus node cells and the excitation of atrial cells excitation [41]. The results of our flow cytometry assay showed that the proportion of Shox2^+^Nkx2-5^+^ cells increased in RA + CHIR = 0 group, while in RA + CHIR = 3 group, Shox2 + Nkx2-5^−^ cells increased considerably. Wnt signaling pathway promotes the formation of structural TBX18^+^ “head” sinus node cells, RA increases the functional “tail” cells, and the combination of both might better mimic the formation of SAN. This further explains the decrease in the beat frequency induced by the overstimulation of Wnt signaling pathway, while the addition of RA can increase and restore the frequency of beats.

RA plays a pleiotropic role during heart development at different concentrations [42]. RA mainly induces epicardial cells at 1-4 µmol/L [43, 44], 0.5-1 µmol/L of RA intervenes the mesoderm to increase the proportion of atrial myocytes [45]. High concentrations of RA (1-100 µmol/L) induce the production and maturation of ventricular muscle cells [46, 47]. Liu and Hou conducted certain experiments [5, 6] and found that RA inhibitors (BMS189453) can increase the proportion of sinus node cells, which appears to contradict our results at first glance. However, a closer analysis shows that BMS453 is an agonist of RARβ receptors, and an inhibitor of RARα as well as RARγ receptors. RA is pleiotropic as a broad agonist of RAR receptors and RXR receptors. After combining our results with those of Liu and Hou, we preliminarily inferred that the promotion of sinus node cell production facilitated by RA might partly be due to the effect on RARβ receptors. Some studies have shown that RA acts upstream of the transcription factor TBX5 and directly regulates the expression of TBX5 [48]. Exogenous RA-induced embryonic stem cells can increase transcriptional levels of Shox2 and ISL-1. These experiments showed that RA plays a key role in the development of SAN, and based on the results of Protze et al., Liu et al. as well as Hou et al., we focused our attention on investigating the RA signaling pathway. In the future, we aim to compare the different effects between BMS189453 and RA or explore the synergistic interaction with other signaling pathways.

The most prominent difference between our experiment and Protze et al. was the time of the intervention. Our experiment initially followed the RA intervention time suggested by the study of Protze et al., but we obtained atrial myocytes following that protocol. Their intervention point was mesoderm cells, but our intervention time was heart mesoderm cells and heart precursor cells, which was consistent with the study by Liu and Hou. Actually, the time of RA intervention in our experiments was consistent with the expression of RA in SAN embryonic development. In the E7.5-E8.0 stage, Raldh2 is initially expressed in the lateral side plate mesoderm, and then came to the pSHF cardiac precursor cells, limited to the sinoatrial node and the atrial area [49]. In order to exclude the specificity of the U1 cells, we induced another cell line (H9) following our protocol, and the results showed that RA can also increase the expression of Shox2 and reduce the mRNA level of Nkx2.5 (Additional file 7: Figure S3). Compared to the proportion of SANLPCs (35%) obtained by Protze et al., our proportion of SANLPCs (21.3%) was lower, but our protocol was more concise. Furthermore, we combined the use of RA in our study with other factors (such as FGFi, BMP4) to determine whether the proportion of SANLPCs can be further increased.

Our experiments had three main limitations: Firstly, the administration of excess RA during pregnancy can have a teratogenic effect on the embryo. RA has a two-sided effect on human gene regulation. Thus, whether it has some potentially detrimental effects (such as carcinogenicity) remains undetermined [50, 51]. Secondly, previous studies have demonstrated that Shox2 acts as an upstream transcription factor of ISL-1 as well as TBX3. According to our results, RA improved the expression of TBX5 and Shox2 but did not affect TBX3 and ISL-1, the reason is not known. That is to say, the biological mechanism by which RA induces sinus node-like cells requires further investigation. Finally, due to the limitations of experimental conditions and taking into account the immunosuppressive effect between species, our experiment ended at the cell stage, and did not include the effects of the administration of RA on animals. Therefore, several animal-related problems should be considered in the follow-up experiments, for example, how to avoid xeno-immune rejection, whether RA has an operative effect in vivo and so on.

## Conclusions

In conclusion, in this study, we found that RA with Wnt signaling can induce hiPSCs to differentiate into sinus node-like cells, increase the proportion of CTNT^+^Shox2^+^Nkx2.5^−^ cells, and rise the frequency of beats. The underlying biological mechanism by which such changes occur might include cooperation with the Wnt signaling pathway to regulate pacing-related transcription factors, such as Shox2, TBX5, Nkx2.5, TBX18 and so on.

## Supplementary Information

**Additional file 1.** Exploration of the optimal RA intervention time.

**Additional file 2.** Exploration of the optimal RA intervention concentration.

**Additional file 3.** Video of group CHIR=0 on D20.

**Additional file 4.** Video of group CHIR=3 on D20.

**Additional file 5.** Video of group RA+CHIR=0 on D20.

**Additional file 6.** Video of group RA+CHIR=3 on D20.

**Additional file 7.** RA promoted the differentiation of hESCs (H9) into pacemaker cells.

## Data Availability

All data generated and analyzed during this study are included in this published article.

## Abbreviations

hiPSCs: Human-induced pluripotent stem cells
RA: Retinoic acid
AP: Action potential
BMP: Bone morphogenic protein
FGF: Fibroblast growth factor
SAN: Sinoatrial node
CMs: Cardiomyocytes
RT-qPCR: Reverse transcription-quantitative polymerase chain reaction
TBP: One of housekeeping gene, TATA-box binding protein
SOX2: SRY-box transcription factor 2
OCT-4: Octamer‐binding protein 4
BryT: Mesoderm-specific T-box transcription factor Brachyury
Mesp1: Mesoderm posterior BHLH transcription factor 1
cTNT: Cardiac troponin T
NPPA: Gene encoding atrial natriuretic peptide
SCN5A: Sodium voltage-gated channel alpha subunit 5
MYL-2: Myosin, light polypeptide 2
TBX5: T-box 5
Nkx2.5: NK2 homeobox 5
Shox2: Short stature homeobox 2
ISL-1: Insulin gene enhancer binding protein 1
HCN4: Hyper-polarization-activated cyclic nucleotide-gated potassium channel 4
Pitx2: Paired-like homeodomain transcription factor 2
KCNJ2: Potassium inwardly rectifying channel subfamily J member 2
Cacna1g: Calcium channel, voltage-dependent, T-type, alpha 1G subunit
NCX: Na-Ca exchanger, cardiac sodium–calcium exchanger
Cx45: Connexins 45
miR-106b: MicroRNA-106b
I_f_: Funny current
APA: Action potential amplitude
APD: Action potential duration
MDP: Maximum diastolic potential
CPCs: Cardiac progenitor cells

## References

[1] Minhas R, Loeffler-Wirth H, Siddiqui YH, Obrebski T, Vashisht S, Abu Nahia K (2021). Transcriptome profile of the sinoatrial ring reveals conserved and novel genetic programs of the zebrafish pacemaker. Bmc Genomics.

[2] Zhang C, Li Y, Cao J, Yu B, Zhang K, Li K (2021). Hedgehog signalling controls sinoatrial node development and atrioventricular cushion formation. Open Biol.

[3] Duong TB, Holowiecki A, Waxman JS (2021). Retinoic acid signaling restricts the size of the first heart field within the anterior lateral plate mesoderm. Dev Biol.

[4] Mossahebi-Mohammadi M, Quan M, Zhang J, Li X. FGF Signaling pathway: a key regulator of stem cell pluripotency. Front Cell Dev Biol. 2020;8. 10.3389/fcell.2020.00079 PMC7040165 32133359

[5] Hou X, Ma S, Fan W, Li F, Xu M, Yang C (2022). Chemically defined and small molecules-based generation of sinoatrial node-like cells. Stem Cell Res Ther.

[6] Liu F, Fang Y, Hou X, Yan Y, Xiao H, Zuo D (2020). Enrichment differentiation of human induced pluripotent stem cells into sinoatrial node-like cells by combined modulation of BMP, FGF, and RA signaling pathways. Stem Cell Res Ther.

[7] Ren J, Han P, Ma X, Farah EN, Bloomekatz J, Zeng XI (2019). Canonical Wnt5b signaling directs outlying Nkx2.5+mesoderm into pacemaker cardiomyocytes. Dev Cell.

[8] Protze SI, Liu J, Nussinovitch U, Ohana L, Backx PH, Gepstein L (2017). Sinoatrial node cardiomyocytes derived from human pluripotent cells function as a biological pacemaker. Nat Biotechnol.

[9] Bernheim S, Meilhac SM (2020). Mesoderm patterning by a dynamic gradient of retinoic acid signalling. Philos Trans R Soc Lond B Biol Sci.

[10] Dominguez JN, Meilhac SM, Bland YS, Buckingham ME, Brown NA (2012). Asymmetric fate of the posterior part of the second heart field results in unexpected left/right contributions to both poles of the heart. Circ Res.

[11] Kawakami Y, Raya A, Raya RM, Rodriguez-Esteban C, Belmonte J (2005). Retinoic acid signalling links left-right asymmetric patterning and bilaterally symmetric somitogenesis in the zebrafish embryo. Nature.

[12] Lian X, Hsiao C, Wilson G, Zhu K, Hazeltine LB, Azarin SM (2012). Robust cardiomyocyte differentiation from human pluripotent stem cells via temporal modulation of canonical Wnt signaling. P Natl Acad Sci Usa.

[13] Zhang W, Zhao H, Quan D, Tang Y, Wang X, Huang C (2022). Tbx18 promoted the conversion of human-induced pluripotent stem cell-derived cardiomyocytes into sinoatrial node-like pacemaker cells. Cell Biol Int.

[14] Niederreither K, Vermot J, Messaddeq N, Schuhbaur B, Chambon P, Dolle P (2001). Embryonic retinoic acid synthesis is essential for heart morphogenesis in the mouse. Development.

[15] Espinoza-Lewis RA, Yu L, He F, Liu H, Tang R, Shi J (2009). Shox2 is essential for the differentiation of cardiac pacemaker cells by repressing Nkx2-5. Dev Biol.

[16] Blaschke RJ, Hahurij ND, Kuijper S, Just S, Wisse LJ, Deissler K (2007). Targeted mutation reveals essential functions of the homeodomain transcription factor Shox2 in sinoatrial and pacemaking development. Circulation.

[17] Liu H, Chen C, Ye W, Espinoza-Lewis RA, Hu X, Zhang Y et al. Phosphorylation of Shox2 Is Required for Its Function to Control Sinoatrial Node Formation. J Am Heart Assoc. 2014;3. 10.1161/JAHA.114.000796 PMC4309068 24847033

[18] Barbuti A, Robinson RB (2015). Stem cell-derived nodal-like cardiomyocytes as a novel pharmacologic tool: insights from sinoatrial node development and function. Pharmacol Rev.

[19] Ionta V, Liang W, Kim EH, Rafie R, Giacomello A, Marban E (2015). SHOX2 overexpression favors differentiation of embryonic stem cells into cardiac pacemaker cells, improving biological pacing ability. Stem Cell Rep.

[20] Kalinderi K, Fragakis N, Koskinas KC, Katritsis D, Letsas K, Efremidis M et al. Variant rs2200733 on chromosome 4q25 independently confers increased risk of atrial fibrillation in a greek population. Cardiovasc Res. 2014;1031.

[21] Mohanty S, Santangeli P, Bai R, Di Biase L, Mohanty P, Pump A (2013). Variant rs2200733 on chromosome 4q25 confers increased risk of atrial fibrillation: evidence from a meta-analysis. J Cardiovasc Electr.

[22] Bellenguez C, Bevan S, Gschwendtner A, Spencer CCA, Burgess AI, Pirinen M (2012). Genome-wide association study identifies a variant in HDAC9 associated with large vessel ischemic stroke. Nat Genet.

[23] Husser D, Adams V, Piorkowski C, Hindricks G, Bollmann A (2010). Chromosome 4q25 variants and atrial fibrillation recurrence after catheter ablation. J Am Coll Cardiol.

[24] Lang D, Glukhov AV. Cellular and molecular mechanisms of functional hierarchy of pacemaker clusters in the sinoatrial node: new insights into sick sinus syndrome. J Cardiovasc Dev Dis. 2021;8. 10.3390/jcdd8040043 PMC8069964 33924321

[25] Thorolfsdottir RB, Sveinbjornsson G, Aegisdottir HM, Benonisdottir S, Stefansdottir L, Ivarsdottir EV (2021). Genetic insight into sick sinus syndrome. Eur Heart J.

[26] Dobrzynski H, Boyett MR, Anderson RH (2007). New insights into pacemaker activity: promoting understanding of sick sinus syndrome. Circulation.

[27] Wang J, Klysik E, Sood S, Johnson RL, Wehrens XH, Martin JF (2010). Pitx2 prevents susceptibility to atrial arrhythmias by inhibiting left-sided pacemaker specification. Proc Natl Acad Sci USA.

[28] De Bono C, Thellier C, Bertrand N, Sturny R, Jullian E, Cortes C (2018). T-box genes and retinoic acid signaling regulate the segregation of arterial and venous pole progenitor cells in the murine second heart field. Hum Mol Genet.

[29] Mori AD, Zhu Y, Vahora Y, Nieman B, Koshiba-Takeuchi K, Davidson L (2007). Tbx5-dependent rheostatic control of cardiac gene expression and morphogenesis (Vol 297, pg 566 2006). Dev Biol.

[30] Raghunathan S, Francisco Islas J, Mistretta B, Iyer D, Shi L, Gunaratne PH (2020). Conversion of human cardiac progenitor cells into cardiac pacemaker-like cells. J Mol Cell Cardiol.

[31] Pezhouman A, Engel JL, Nguyen NB, Skelton RJP, Gilmore WB, Qiao R et al. Isolation and characterization of hESC-derived heart field-specific cardiomyocytes unravels new insights into their transcriptional and electrophysiological profiles. Cardiovasc Res. 2021. 10.1093/cvr/cvab102 PMC9020202 33744937

[32] Burnicka-Turek O, Broman M, Steimle JD, Boukens B, Petrenko NB, Ikegami K et al. Transcriptional patterning of the ventricular cardiac conduction system. Circ Res. 2020;1271. 10.1161/CIRCRESAHA.118.314460 PMC8328577 32290757

[33] Rathjens FS, Blenkle A, Iyer LM, Renger A, Syeda F, Noack C (2021). Preclinical evidence for the therapeutic value of TBX5 normalization in arrhythmia control. Cardiovasc Res.

[34] Bressan M, Liu G, Mikawa T (2013). Early mesodermal cues assign avian cardiac pacemaker fate potential in a tertiary heart field. Science.

[35] Liang W, Han P, Kim EH, Mak J, Zhang R, Torrente AG (2020). Canonical Wnt signaling promotes pacemaker cell specification of cardiac mesodermal cells derived from mouse and human embryonic stem cells. Stem Cells.

[36] Zhao M, Tang Y, Zhou Y, Zhang J. Deciphering Role of Wnt Signalling in Cardiac Mesoderm and Cardiomyocyte Differentiation from Human iPSCs: Four-dimensional control of Wnt pathway for hiPSC-CMs differentiation. Sci Rep-Uk. 2019;9. 10.1038/s41598-019-55620-x PMC6920374 31852937

[37] Liang D, Xue Z, Xue J, Xie D, Xiong K, Zhou H (2021). Sinoatrial node pacemaker cells share dominant biological properties with glutamatergic neurons. Protein Cell.

[38] Tang W, Li Y, Li A, Bronner ME. Clonal analysis and dynamic imaging identify multipotency of individual Gallus gallus caudal hindbrain neural crest cells toward cardiac and enteric fates. Nat Commun. 2021;12. 10.1038/s41467-021-22146-8 PMC7994390 33767165

[39] Bressan M, Henley T, Louie JD, Liu G, Christodoulou D, Bai X (2018). Dynamic cellular integration drives functional assembly of the heart's pacemaker complex. Cell Rep.

[40] Wiese C, Grieskamp T, Airik R, Mommersteeg MT, Gardiwal A, de Gier-de VC (2009). Formation of the sinus node head and differentiation of sinus node myocardium are independently regulated by Tbx18 and Tbx3. Circ Res.

[41] Li H, Li D, Wang Y, Huang Z, Xu J, Yang T (2019). Nkx2–5 defines a subpopulation of pacemaker cells and is essential for the physiological function of the sinoatrial node in mice. Development.

[42] Wiesinger A, Boink G, Christoffels VM, Devalla HD (2021). Retinoic acid signaling in heart development: application in the differentiation of cardiovascular lineages from human pluripotent stem cells. Stem Cell Rep.

[43] Guadix JA, Orlova VV, Giacomelli E, Bellin M, Ribeiro MC, Mummery CL (2017). Human pluripotent stem cell differentiation into functional epicardial progenitor cells. Stem Cell Rep.

[44] Tan JJ, Guyette JP, Miki K, Xiao L, Kaur G, Wu T (2021). Human iPS-derived pre-epicardial cells direct cardiomyocyte aggregation expansion and organization in vitro. Nat Commun.

[45] Cyganek L, Tiburcy M, Sekeres K, Gerstenberg K, Bohnenberger H, Lenz C (2018). Deep phenotyping of human induced pluripotent stem cell-derived atrial and ventricular cardiomyocytes. JCI Insight.

[46] Wobus AM, Kaomei G, Shan J, Wellner MC, Rohwedel J, Ji G (1997). Retinoic acid accelerates embryonic stem cell-derived cardiac differentiation and enhances development of ventricular cardiomyocytes. J Mol Cell Cardiol.

[47] Miao S, Zhao D, Wang X, Ni X, Fang X, Yu M (2020). Retinoic acid promotes metabolic maturation of human embryonic stem cell-derived cardiomyocytes. Theranostics.

[48] Feneck E, Logan M (2020). The role of retinoic acid in establishing the early limb bud. Biomolecules.

[49] Ivanovitch K, Soro-Barrio P, Chakravarty P, Jones RA, Bell DM, Mousavy GS (2021). Ventricular, atrial, and outflow tract heart progenitors arise from spatially and molecularly distinct regions of the primitive streak. Plos Biol.

[50] Bittner M, Stern A, Smutna M, Hilscherova K, Zegura B (2021). Cytotoxic and genotoxic effects of cyanobacterial and algal extracts-microcystin and retinoic acid content. Toxins.

[51] Shiah S, Hsiao J, Chang H, Hsu Y, Wu G, Peng H (2020). MiR-30a and miR-379 modulate retinoic acid pathway by targeting DNA methyltransferase 3B in oral cancer. J Biomed Sci.

