# Supplementary figures and images for: RA signaling pathway combined with Wnt signaling pathway regulates human-induced pluripotent stem cells (hiPSCs) differentiation to sinus node-like cells
Source: Stem Cell Res Ther. 2022 Jul 18;13:324. doi: 10.1186/s13287-022-03006-8 (PMC9290266; doi:10.1186/s13287-022-03006-8)

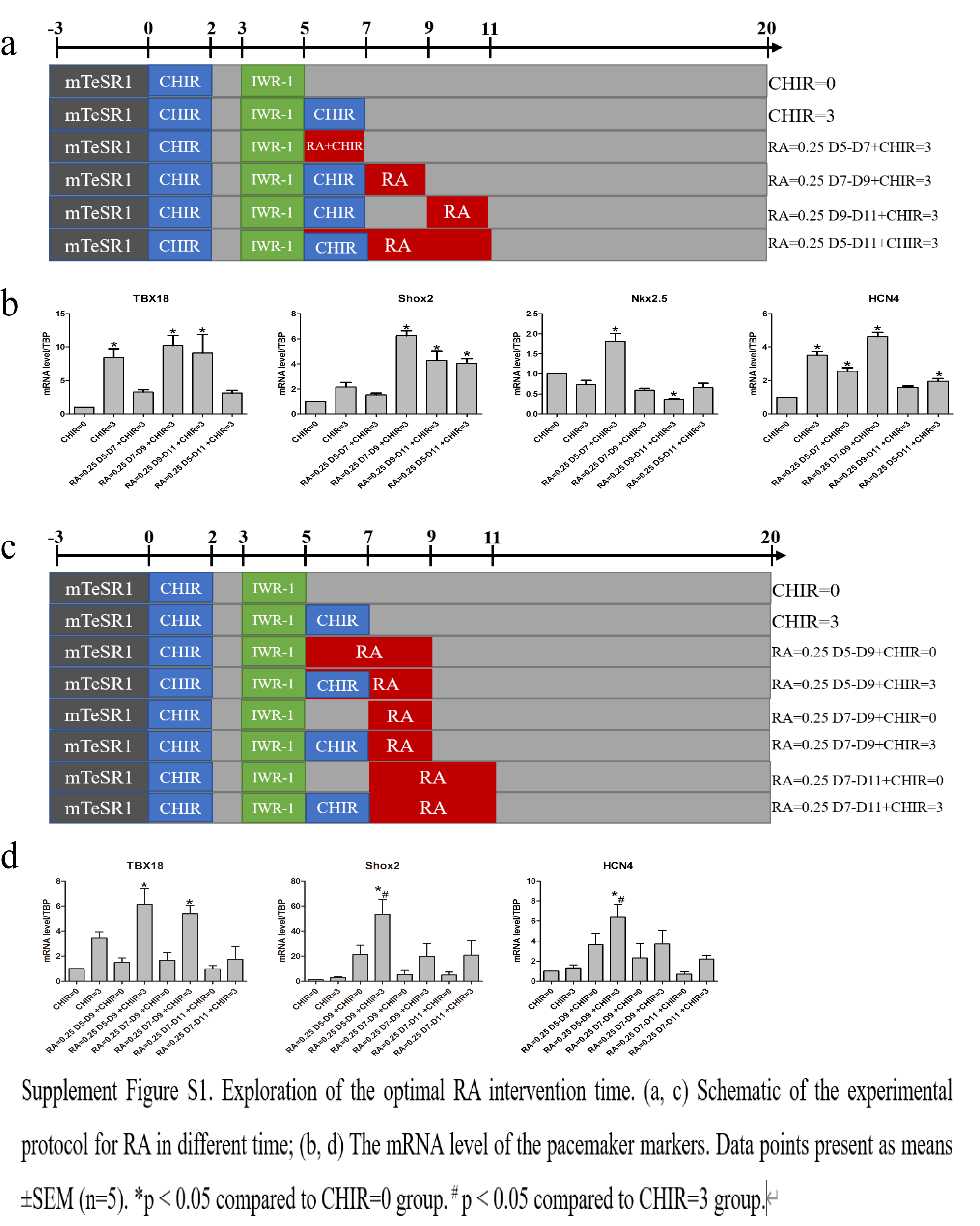

Supplement: Supplementary file 1 — Additional file 1. Exploration of the optimal RA intervention time. [file 13287_2022_3006_MOESM1_ESM.tif]

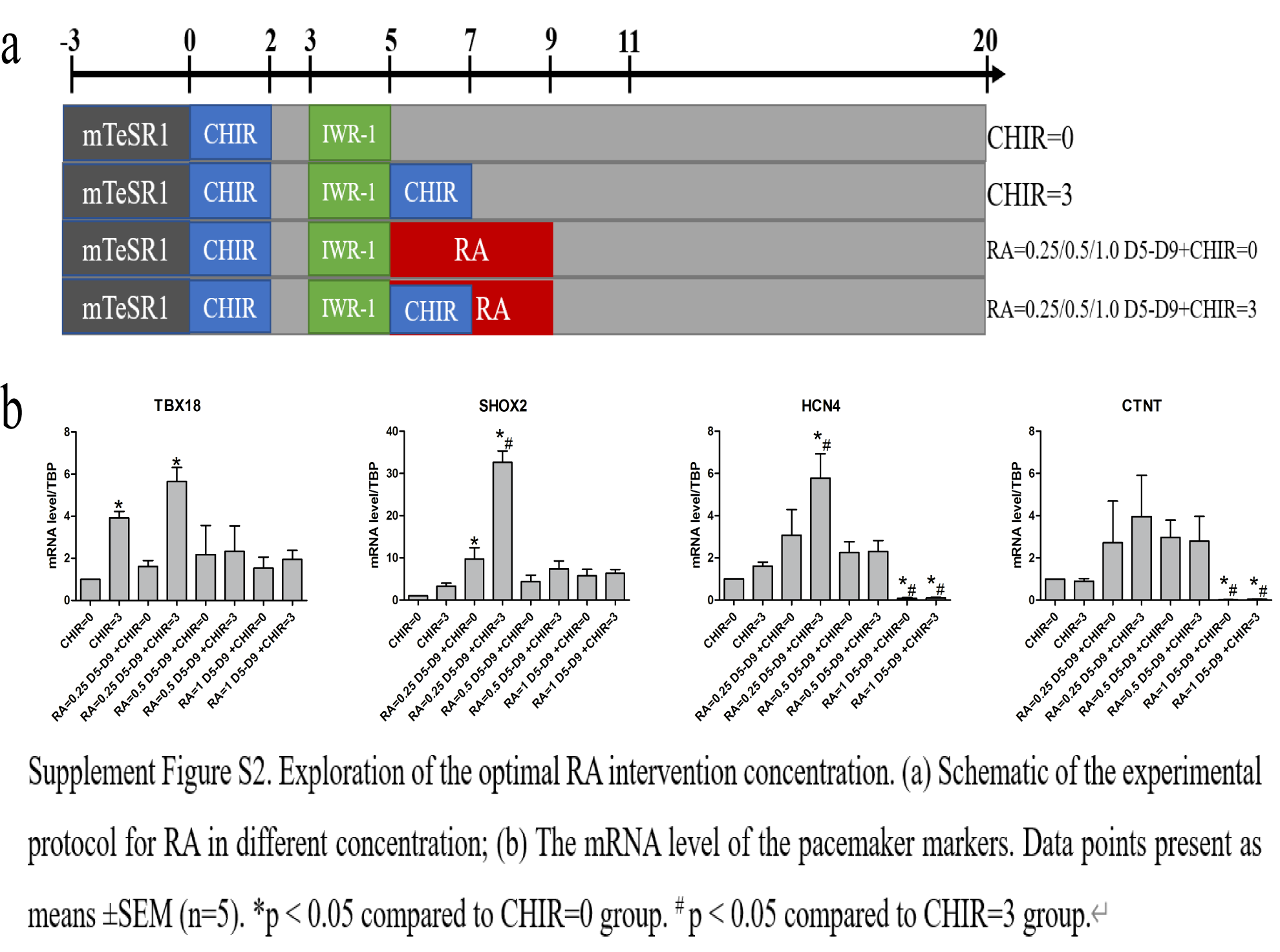

Supplement: Supplementary file 2 — Additional file 2. Exploration of the optimal RA intervention concentration. [file 13287_2022_3006_MOESM2_ESM.tif]

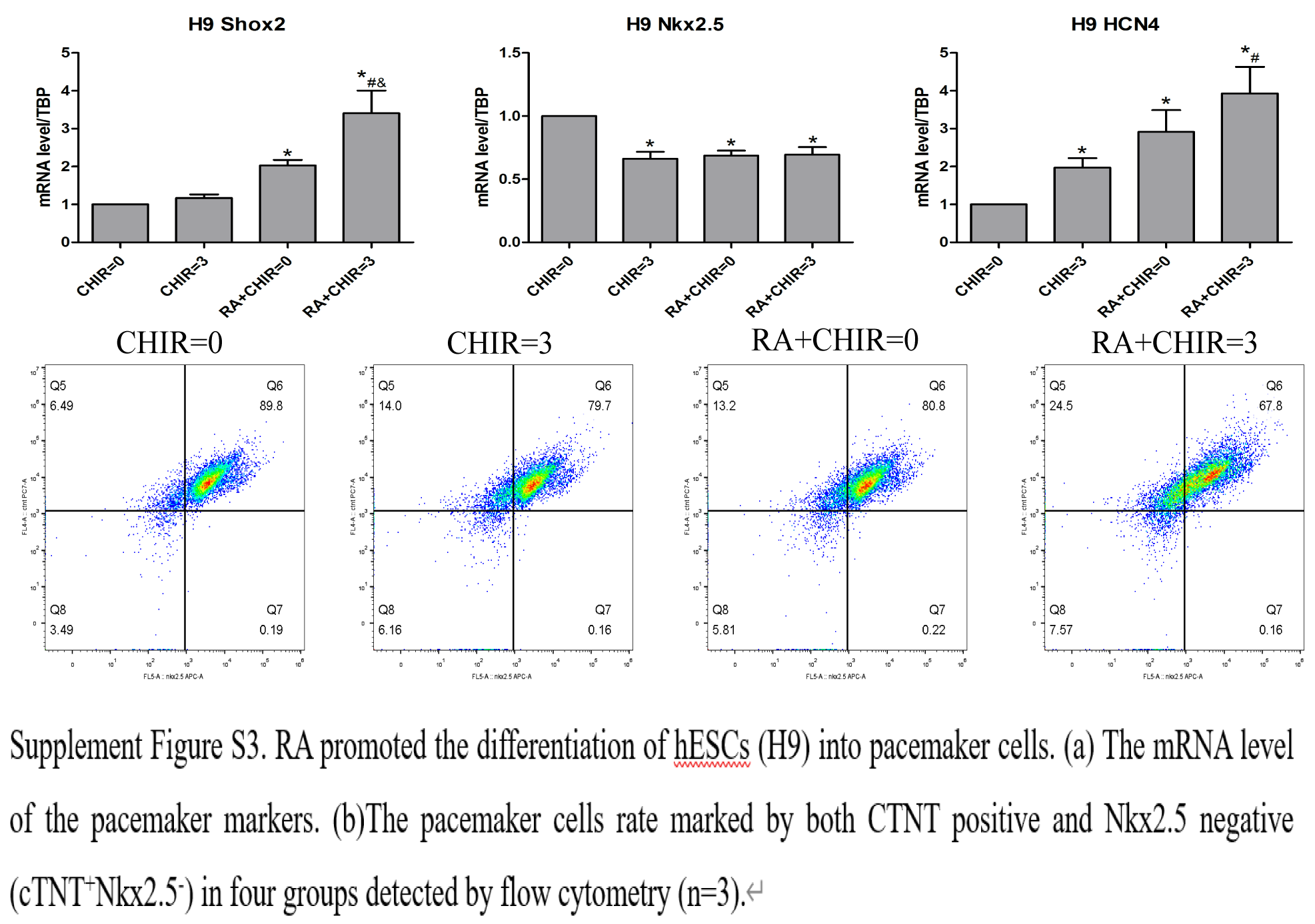

Supplement: Supplementary file 7 — Additional file 7. RA promoted the differentiation of hESCs (H9) into pacemaker cells. [file 13287_2022_3006_MOESM7_ESM.tif]
